# Bangladeshi Native Vehicle Classification Based on Transfer Learning with Deep Convolutional Neural Network

**DOI:** 10.3390/s21227545

**Published:** 2021-11-13

**Authors:** Md Mahibul Hasan, Zhijie Wang, Muhammad Ather Iqbal Hussain, Kaniz Fatima

**Affiliations:** 1 College of Information Science and Technology, Donghua University, Shanghai 201620, China; mmhasan.ete@gmail.com (M.M.H.); 415030@mail.dhu.edu.cn (M.A.I.H.); 2 Institute of Business Administration, Jahangirnagar University, Savar, Dhaka 1342, Bangladesh; kanizulc@gmail.com

**Keywords:** native vehicle type classification, Deshi-BD vehicle dataset, deep learning, transfer learning, ResNet-50

## Abstract

Vehicle type classification plays an essential role in developing an intelligent transportation system (ITS). Based on the modern accomplishments of deep learning (DL) on image classification, we proposed a model based on transfer learning, incorporating data augmentation, for the recognition and classification of Bangladeshi native vehicle types. An extensive dataset of Bangladeshi native vehicles, encompassing 10,440 images, was developed. Here, the images are categorized into 13 common vehicle classes in Bangladesh. The method utilized was a residual network (ResNet-50)-based model, with extra classification blocks added to improve performance. Here, vehicle type features were automatically extracted and categorized. While conducting the analysis, a variety of metrics was used for the evaluation, including accuracy, precision, recall, and $F_{1}\;-\;\mathrm{Score}$. In spite of the changing physical properties of the vehicles, the proposed model achieved progressive accuracy. Our proposed method surpasses the existing baseline method as well as two pre-trained DL approaches, AlexNet and VGG-16. Based on result comparisons, we have seen that, in the classification of Bangladeshi native vehicle types, our suggested ResNet-50 pre-trained model achieves an accuracy of 98.00%.

## 1. Introduction

Road traffic accidents are a global concern due to the increasing amount of people who die, or are extremely injured, because of these accidents. Statistics show that, each year, around 1.2 million people die as a result of road incidents. Moreover, statistics show that over 50 million people are injured in road accidents globally [1]. However, the phenomenon varies by country. When compared with developed countries, the number of injuries, deaths, and accidents are 10 to 60 times higher in developing nations [2]. Over 80% of total road injuries take place in the world’s developing regions [3]. Bangladesh is one of a few developing nations where the rate of injuries, deaths, and accidents is extremely high. The current situation is worse than ever before. For example, 20 people on average die each day because of road accidents. As per the guidelines from the United Nations Road Safety Action Plan 2011–2020, the Sustainable Development Goals (SDGs) 2030, and the associated GOAL-3.6, Bangladesh is required to cut the number of road traffic injuries and deaths in half [3,4]. Considering this and keeping in harmony with developed countries, it is crucial for Bangladesh to be dependent on an intelligent transportation system, to develop its traffic management system.

An intelligent transportation system (ITS) is a widely used term, related to the concept used in road and transportation planning. The aim is to enhance the performance and security of, for example, superhighway tolls, traffic counts, and traffic observations. One outstanding feature of an ITS is vehicle type classification. It offers a broad range of applications, including smart parking solutions, traffic management statistics, and identification of vehicle types. Prevailing strategies rely on ultrasonic, sensors, and video devices. The computer vision (CV) community is focusing on image-based methods due to the extensive utilization of vehicle monitoring devices. From the perspective of an ITS, object classification of vehicles plays an essential role. It has an extensive variety of engagement, consisting of traffic monitoring, routing, and tracking. Object classification involves an enormous discipline of research in regard to image processing techniques and it seeks to classify elements in images, into significant groups [5]. Individuals can categorize vehicles, without difficulty, from images via key aspects, such as trademarks, forms, and ornaments. Nonetheless, classification of vehicle types in images is, perhaps, a tough issue for computer systems. This is due to the fact that image sources have multi-scale characteristics [5]. Vehicles are also available in a wide range of shapes, measurements, and shades. Furthermore, natural factors, such as illumination, noise, complex background, and climate, affect the ability to capture photos in traffic.

For vehicle type classification [6,7,8,9], techniques based on laser and loop induction detectors have already been presented. In these methods, with the aim to collect and analyze records and bring out relevant information regarding vehicles, the sensors were installed under road pavements. However, adverse weather and damage in walkways are responsible for influencing the correctness and stability of these methodologies [10]. CV has progressed tremendously in recent years. Thus, the authors have suggested the use of vehicle classification systems, which are based on pattern identification and image analysis [11,12]. This is actually a process consisting of two stages. The first stage incorporates visual characteristics from input visual frames using handcrafted extraction methods. In the second stage, training on the extracted features is provided to the machine learning (ML) classifiers, in order to categorize data, depending on the types. Moreover, there are two types of customized characteristics: (i) global and (ii) local. The division helps to define and categorize the image information [13]. All of these attributes are used to train existing ML classifiers. Consequently, they help with object classification. The mentioned methods perform very well in specific regulated surroundings. Moreover, these systems are more convenient to install (and look after) as compared to existing methods, which are dependent on laser and inductive processes. However, the algorithms are given training on insufficient customized characteristics derived from limited datasets, but maintaining accuracy in a temporal setting requires significant prior information [14].

Deep learning (DL)-based feature extraction and classification approaches have recently gained popularity, exhibiting superior adaptability and flexibility over conventional methods. Because of their improved design, CNN classification algorithms gained notable precision on wide-range databases [15,16,17]. To date, as far as we know, for the creation and application of Bangladeshi vehicle classification systems, there is no universal benchmark dataset available. The existing vehicle classification datasets, such as CompCars [18] and Stanford Cars dataset [19], are very small. These are based on narrow classifications of certain locations. Encouraged by the prior studies and the enthusiasm to address the limitations, this paper will focus on the vehicle type classification on roads, so that various nations, especially South Asian nations (e.g., Bangladesh), could take advantage from its implementation. These countries are still using conventional strategies manually observed via human, photography, and sensor-based programs. Thus, a competent approach of the traffic surveillance system in Bangladesh is required to acquire accurate final results. By applying data augmentation and transfer learning approaches, we present a DL model for recognition and classification of Bangladeshi native vehicles.

To overcome all of the above problems in Bangladeshi native vehicle classification systems, we established the below-mentioned improvements to our native vehicle classification system.

(i). We introduced a Bangladeshi native vehicle dataset, the Deshi-BD dataset, which contains 10,440 images based on 13 Bangladeshi on-road vehicle classes. These images were manually collected from driving videos by us. It is important to note that these classes are distinct, in terms of features and shapes, and they are not limited in the current datasets.
(ii). A pre-trained CNN model using ResNet-50 [20] was implemented to increase the flexibility of Bangladeshi native vehicle classification techniques under poor illuminating circumstances.
(iii). We evaluated various performance measures for our native vehicle classification model, including (i) accuracy; (ii) precision; (iii) recall; and (iv) $F_{1}\;-\;\mathrm{Score}$. We also compared our suggested approach with AlexNet and VGG-16 CNN models that have been trained.
(iv). Finally, an overall comparison of the anticipated and standing vehicle classification techniques were performed to present an accurate possibility of our native vehicle classification network proposal. As seen by the results, our suggested technique provides greater classification precision when compared to other traditional methods.

The following is how we structured our paper. We present several similar studies and emphasize their findings in Section 2. We explain the proposed approach in Section 3. We provide the analysis of results in Section 4. In Section 5, we offer a discussion and a comparison with previous works. Finally, in Section 6, we provide a conclusion and suggestions for further work.

## 2. Related Work

Artificial intelligence is developing at a high speed. Accordingly, vision-based vehicle classification is regarded as a critical component of driverless vehicle observation units. The two primary forms of vision-based vehicle classification techniques are the (i) customized feature-based technique and (ii) deep feature-based technique [10]. These groupings are found in existing research works. During the primary period of CV, customized feature-based approaches were expected for ITSs. Researchers used the HOG-SVM based customized features approach for training a SVM classifier utilizing HOG features along with the Gaussian Kernel feature suggested by Ng et al. [21]. The aforementioned classification model was tested on a surveillance footage collection of 2800 images. It classified three types of vehicles with 92.3% correctness. Wen et al. [22] conducted the study, applying an AdaBoost-based rapid learning vehicle predictor to separate data, which were categorized as (i) non-vehicle and (ii) vehicle. 

Furthermore, for the rapid learning of classifiers, the authors suggested a procedure for generating Haar-like attributes. This current classifier was tested for an open Caltech data source and gained correctness of 92.89%. Matos et al. [23] presented a combined method for integrating vehicle characteristics such as width, height, enclosing lines, etc. These were based on two neural networks. This suggested architecture, having a sample size of 100, accomplished 69% correctness. On the other hand, a classification method was demonstrated by Chen et al. [24] that extracted both texture and HOG attributes. It also classified the vehicles by utilizing a fuzzy enthused SVM classification model. The demonstrated classifier achieved accuracy of 92.6%. It was evaluated on a dataset having 2000 images of cars, vans, and buses. A collective method, integrating the SIFT classifier and BoW-based method, was suggested by Cui et al. [25] with the aim to extract the characteristics and apply SVM to classify the dataset. The dataset was a group of 340 images of trucks, cars, and minibuses. The ultimate result achieved a 90.2% accuracy by the presented classifier.

Moreover, deep feature-based systems can mitigate the issues related to handcrafted feature-based classifiers. A CNN-based semi supervised classification system for simultaneous vehicle classification was demonstrated by Dong et al. [26]. In their work, a sparse-Laplacian filter-dependent process was planned to extract comparative vehicle data. Moreover, to compute the class likelihood of the associated vehicle, a softmax layer was trained. They evaluated the data using the Bit-Vehicle database, where day scenes demonstrated 96.1% correctness and night scenes demonstrated 89.6% correctness. For vehicle sorting in an uncontrolled road atmosphere, a CNN and an end-to-end combined model were suggested by Cao et al. [27]. The expected structure succeeded in providing a 95.3% correctness, evaluated on the CompCars view-aware dataset.

Moreover, Jo et al. [28] established GoogLeNet architecture that focused on transfer learning. It was used for vehicle classification of road traffic; the classifier achieved 98.3% accuracy. The research was based on the ILSVRC-2012 dataset. The classification and identification of vehicles on highway routes has a strong influence on traffic and accident management. CNN architecture, which focuses on the vehicle classification algorithm for vehicle classification and numbering on major routes, was an idea from Chauhan et al. [29]. After applying 5562 CCTV camera videos on highways, they demanded that their offered model achieved 75% MAP. In the research work by Kim et al. [30], they used the PCANeT-HOG-HU model that focuses on the collective characteristic extracting procedure. In this case, the methodology was fed into SVM as input data for training the classifier architecture. The authors extracted 13,700 images of cars from surveillance recordings for training and testing the suggested classifiers model. The dataset comprised six types of vehicles. They suggested mild-mass classification architecture, which obtained a mean correctness of 98.34%. Fast R–CNN architecture, which focuses on vehicle classification techniques, was suggested by Wang et al. [31]. The aim was to develop a technique for traffic surveillance in a real-time atmosphere. The authors collected and tested a sample with 60,000 images. These data were gathered and separated to train and test sets. The total correct result obtained was 80.051%.

Other researchers have proposed a method, focusing on deep features, which could significantly improve vehicle recognition accuracy. However, they require a large amount of data to achieve considerable precision in the present ITS operations [32,33,34,35]. In the modern era, extensive research has been conducted in this field; yet, the current civic resources for vehicles or ITSs include automotive kinds. These are common in wealthy nations. The classification methods, however, are unrealistic regarding ITSs in South Asian nations. All identified difficulties point to the need for a unique vehicle classification methodology, as well as a collection that includes common vehicles, such as conventional buses, trucks, cars, CNG, motorbikes, rickshaws, auto rickshaws, and vans in South Asian nations.

## 3. Materials and Methods

**Figure 1:**
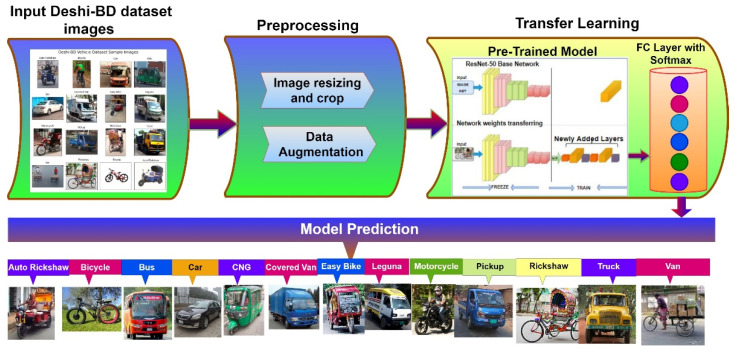
The proposed method.

To solve the above-mentioned problems, we propose a new vehicle dataset consisting of 10,440 images of Bangladeshi typical traffic vehicles, divided into thirteen types. To improve the performance of the recommended classification in real-time ITS applications, the ultimate system was customized using existing AlexNet [36], VGG [37], and ResNet [20] models. Depending on the performance of these models, the top performing model was implemented to improve the accuracy of our system. As a result, we saw that ResNet surpasses the other models in terms of closure, accuracy, and processing speed. Therefore, the ResNet model with 50 layers was improved and implemented in our proposed vehicle classification system. The proposed method is described in detail in Figure 1.

### 3.1. Deshi-BD Vehicle Dataset

**Figure 2:**
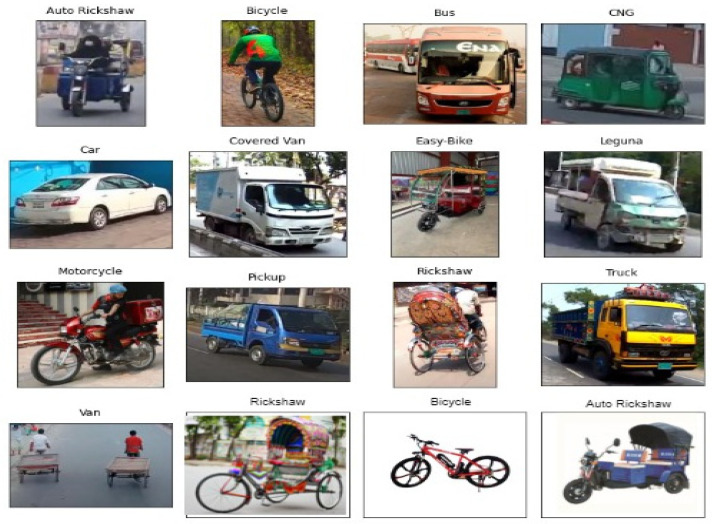
Sample Deshi-BD dataset images representing each class.

The data source is an important component in supporting algorithms in learning features and making predictions based on the learned information when using DL based classification systems. To the best of our knowledge, there seems to be no standardized Bangladeshi general vehicle dataset that includes data on Bangladeshi native vehicles and that solves classification problems. As documented, CompCars as well as the Stanford vehicle database simply incorporate the types of modern vehicles in specific areas. These cannot be used in actual-time classifiers in other geographies, such as Bangladesh. In addition, the recommended data source differs from the available data sources. There is a lot of variation regarding characteristics and structure. Furthermore, the present vehicle classifier may not execute well enough in practical ITS implementations, as it is developed on fairly short data samples with few classes [38]. To address these issues, we created a Bangladeshi native vehicle database with 10,440 images, divided into 13 categories. Figure 2 presents the sample dataset images for each class.

For this dataset, road surveillance and driving videos were gathered from Bangladeshi transportations and highway roads in various weather conditions, such as daylight, foggy day, and rainy day, and different lighting conditions, such as sunny, low light, and dark (in the night) conditions, to properly extract the required images. In this study, thirteen native Bangladeshi vehicle types were determined. The sizes of the objects in the collected images range from very large objects, such as trucks, to little objects, such as traffic plants that are difficult to detect with high accuracy [39]. After collecting the images, the database was created by hand labeling with the help of a Windows snipping editing application. The goal of creating this particular dataset is to build and analyze the collected Bangladeshi traffic images. It will be used to increase our systems acceptability to enhance ITS in South Asian nations, such as Bangladesh. The dataset contains 10,440 pictures, which are classified into thirteen categories (auto rickshaw, bicycle, bus, car, CNG, cover van, easy bike, leguna, motorcycle, pickup, rickshaw, truck, and van). Figure 2 presents some sample images in our Deshi-BD dataset and Table 1 shows image detail information of the dataset. Data augmentation was applied on a low number of image classes to make our dataset more robust.

### 3.2. Data Preprocessing

Data preprocessing is critical to complete a deep learning-based classifier, such as vehicle classification. This is because the vehicle images are collected from various sources and, thus, data preprocessing is done to remove noise or unwanted background, to resize the standard format image, and to make the vehicle images having uneven lighting system. The preprocessing stage is divided into three separate parts:

i. Noisy/mislabeled vehicle image elimination;
ii. Vehicle images resizing;
iii. Augmentation.

#### 3.2.1. Noisy/Mislabeled Vehicle Image Elimination

This section is explored unwanted background, noisy/mislabeled vehicles that will reduce the accuracy of prediction. First, we manually removed the noisy/mislabeled vehicle images from our Deshi-BD vehicle dataset. Images from our Deshi-BD vehicle dataset were categorized as (i) positive and (ii) negative for each class. In this way, Bangladeshi native vehicle pictures can be ensured. Additionally, we could ensure the model’s efficiency, classified as positive and negative. Because the datum source was limited, data augmentation methods were applied to increase test pictures and, therefore, enhance productivity of DL architecture while avoiding overfitting issues.

#### 3.2.2. Vehicle Images Resizing

Because data in the ImageNet dataset differ in terms of size, we created a standard size for all images input into our DL model, and decreased resolution to “save” the preparation and simulation period, as these systems must be evaluated for video classification.

#### 3.2.3. Data Augmentation

When dealing with classification models, such as vehicle classification based on CNN and DL architecture, it is essential to process image data. To address the issue of the limited training data size, data augmentation was used [40]. This technique executes some manipulations on the whole dataset. The goal was to create a collection of varied scenes, therefore expanding the data. The DL method accomplishes perfect results in case of larger datasets. Data augmentation is also used on drone image datasets to improve the accuracy of object identification and ensemble models [39]. We applied augmentation to increase the total images in our dataset. This resulted in permitting the model to train successfully. Data augmentation is a strategy for making the entire database more robust. As a result, by extending the dataset, the method decreases overfitting and improves generalization ability. Here, what is most serious is the second issue. Data augmentation solves the problem without causing any changes to the model’s structure. However, Bangladeshi native vehicle picture collections are few. They are also challenging to obtain during the COVID-19 pandemic.

**Figure 3:**
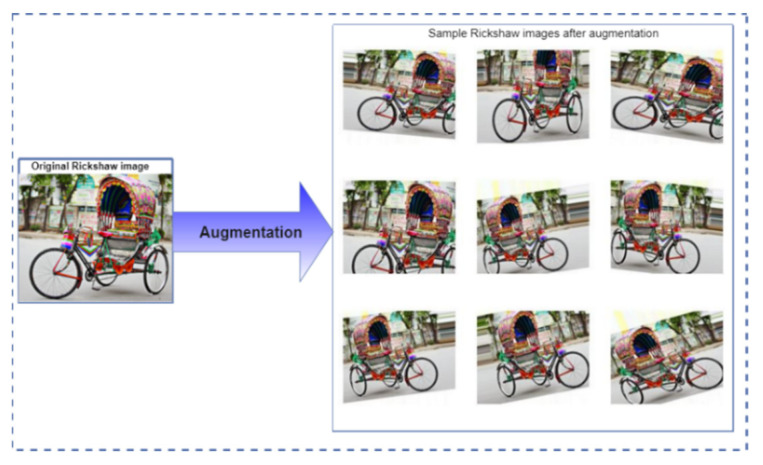
Techniques of data augmentation performed on sample images.

By artificially boosting the sample using label-preserving modification algorithms, parametric data augmentation is the simplest and most frequent way to overcome the problem of model overfitting [41]. We used a multiple augmentation approach with the vehicle images to improve the variation of our sample: (i) rotation, (ii) horizontal flip, (iii) shifting (width shift and height shift), (iv) zooming, (v) brightness adjustment, and (vi) shearing. Figure 3 shows a diagram of these improved views. The data augmentation phase aids in the development of a robust native vehicle classifier utilizing minimal training information and improves the efficiency of the DL algorithm. These augmentation methods are related to the real life scenario.

### 3.3. Convolutional Neural Network (CNN) Model

CNN is a well-known cutting-edge neural network technology that is useful in CV tasks [42]. CNN is a type of deep neural network that filters inputs for meaningful information using convolutional layers. CNN’s convolutional layers apply convolutional filters to the input to compute the output of neurons connected to particular areas in the input. It is useful for extracting spatial and temporal characteristics from images. In CNN’s convolutional layers, a weight-sharing mechanism is implemented to reduce the total number of parameters [43,44]. CNN is usually made up of three layers: (i) a convolutional layer for learning spatial and temporal features; (ii) pooling (a subsampling) layer for reducing or subsampling the dimensionality of an input image; and (iii) a fully connected (FC) layer for classifying the input image into various classes.

#### 3.3.1. Transfer Learning

**Figure 4:**
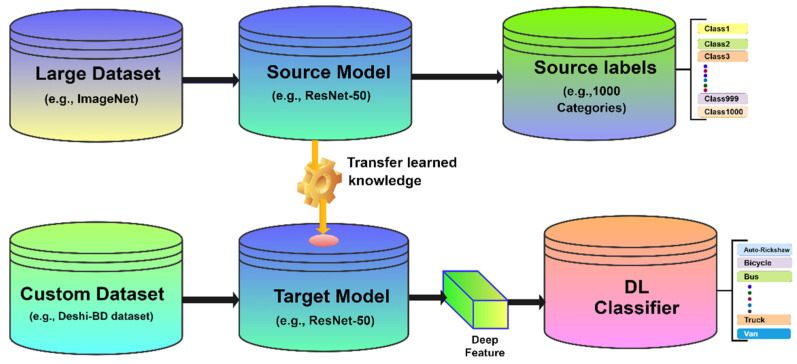
Transfer learning concept.

Transfer learning has been used successfully in a variety of applications, including vehicle image classification and segmentation, in recent years. Transfer learning allows us to learn a generic classifier, using a large amount of labeled data from the source domain and a small amount of labeled data from the destination domain in classification problems. In general, CNN performs better in large datasets than in smaller ones. When it is not possible to create a large training dataset, transfer learning can be used. Figure 4 shows the concept of transfer learning, where a model pre-trained on large benchmark datasets may be utilized as a feature extractor for a new role, using a relatively custom dataset, such as a Deshi-BD dataset.

In this research, transfer learning is used to solve the challenge of determining the classes of Bangladeshi native vehicle images. Because of the size and complexity of CNN architectures, developing and testing models could be costly and time-consuming. When addressing a vehicle type classification, a technique called transfer learning can give faster and more efficient outcomes. In transfer learning, weights, and convolutional filters that are capable of one task (learned for any classification job) could be reused for another task that requires just a little bit of retraining and can be learned or evaluated on a limited number of images. Using a pre-trained neural network model with pre-loaded weights, adjusting it to some amount, and then retraining part or the entire model to fulfil the new task are examples of this. The filters trained by one task are used to extract features from images, which are then interpreted by the retrained component of the neural network in order for it to complete its new task. In this research, the deep convolutional neural network, known as ResNet [20], is used to examine transfer learning, utilizing pre-training over the “ImageNet” dataset [45], and the weights are the same as in ResNet [20].

Transfer learning is performed by replacing the final few layers of the original network, including the output layer, with new fully connected layers that are appropriate for the new challenge. There are two methods to use transfer learning from a model: reuse the model as a feature extractor and apply a completely different classifier, or reuse the model to do fine-tuning. Fine-tuning is an approach that uses unfrozen layers of a complete model to slightly change both the new fully connected layers of the classifier and specific CNN layers, such as convolutional layers [46]. Transfer learning has started from fully connected layer because of a fully connected (FC) layer for classifying the input image into our target classes.

#### 3.3.2. Convolutional Layer

This is the initial layer and one of the core parts of a convolutional neural network (CNN). Definite sets of learnable filters are present in this layer, regarded as a supreme layer in the CNNs. Spatially—input-sizes are greater than the filters. During the forward pass, these filters slip across the input feature to produce a 2D feature map that indicates location as well as the strength of the identified visual elements in data source. The following formula is used to calculate the characteristics of these layers:

(1) $$y_{n}^{l}=fl\left(\sum m⟶\begin{array}{c} l\\ n \end{array}y_{m}^{l-1}\right)$$

Here, the $n^{th}\;$ feature map of l-layer is $\;y_{n}^{l}\;$, C-kernel is $\;m⟶\begin{array}{c} l\\ n \end{array}\;$, while feature extraction from layer-l and $y_{m}^{l-1}$ is the characteristic pattern connected to layer-l.

#### 3.3.3. Pooling Layer

Pooling layers use the image building connection concept to execute pooling procedures on feature maps, in order to reduce network congestion while holding the key characteristics. They are commonly utilized among the CNN model consecutive convolutional layers, and are performed to slowly reduce the spatial display space, decreasing operations while preserving crucial data, which helps in minimizing overfitting during the training process.

Average pooling: as the filter moves over the input, it calculates the average value inside the receptive field to send to the output array.

Maximum pooling: as the filter passes over the input, it picks the pixel with the highest value to transfer to the output array. As an aside, this technique is more commonly used than average pooling. The pooling function can be implemented through:

(2) $$y_{n}^{l}=fl\left(z_{n}^{l-1}xw_{n}^{l}+b_{n}^{l}\right)$$

Here, $z_{n}^{l-1}\;$ value is extracted from l−1 convolution features, $w_{x}^{l}$ represents map weight, and $b_{x}^{l}$ represents offset value.

#### 3.3.4. Dropout Layer

While training the CNN model, we observed a significant amount of overfitting. Thus, to lessen the influence of overfitting, the dropout layer was deployed. Another popular approach used in CNN, to sidestep consequences of overfitting, is regularization. This is accomplished by applying a substantial charge to the loss function in use. Hence, the particular layer is included in the final recommended system, which aids in preventing the system from becoming dependent on the other feature weights.

#### 3.3.5. Fully Connected Layer

The conventional method for image classification problems is to utilize a stack of fully-connected layers, followed by a softmax activated layer [37]. The probability distribution over each possible class label is generated by the softmax layer, and then we simply classify the image based on the most possible class. The last part of the CNN architecture is the fully connected layer, after the two stages of alternate convolution, batch normalization, ReLU, and pooling sublayers. In order to minimalize the feature dimensions, neurons inside this layer are linked to every activation in the former layer. These layers are essentially just like a normal neural network. Here, they map the flattened data into the class labels and generate values for each output variable. Eventually, overall the results of these layers are supplied as inputs for a softmax layer, where the values are transformed, ranging between 0 to 1 and ending in a total of 1. In this approach, the softmax layer depicts the result as a real probability. Fully connected neurons can be described mathematically as follows:

(3) $$y_{n}^{l}=fl\left(\sum_{m=1}^{N_{l-1}}y_{m}^{l-1}\;w_{m,n}^{l}+b_{n}^{l}\right)$$

Here, $N_{l}$ represents value of neurons of output layer, $y_{m}^{l-1}\;$ represents m characteristic pattern of layer  l−1, and $w_{m,n}^{l}$ represents connected weights.

### 3.4. AlexNet

For the first time, AlexNet was introduced in 2012, which used an eight-layer CNN model. This model won the ImageNet Large Scale Visual Recognition Competition by an extraordinarily great margin. AlexNet demonstrated how learning-derived features might outperform manually generated features, shattering the prior CV standard. Although AlexNet [36] is a commonly used deep CNN network, it may still achieve viable classification efficiency when compared to other types of networks. During this model’s training step, the input data are scaled to 224 × 224 pixels and fed into the system. The AlexNet design initially utilizes a convolutional layer to conduct convolutional as well as max pooling through local response normalization utilizing 96 distinct size 11 × 11 receptive filters. Max-pooling activities were carried out using 3 × 3 filters with a stride size of 2. Similar processes were carried out in the second layer with 5 × 5 filters. Moreover, 3 × 3 filters were utilized, as well as fourth and fifth convolutional layers with 384, 384, and 296 feature maps, correspondingly. The output of the two fully linked layers is utilized as a feature extraction vector with dropout, then a softmax layer at the finish point.

### 3.5. VGGNet

VGG-16 is a CNN model [37] with thirteen convolutional layers and three fully-connected layers for a total of 16 weight layers. More exactly, the size of VGG-16 trained ImageNet weights is 528 MB, and VGG16 contains 138 million parameters in total. Therefore, it takes quite a lot of disk space and bandwidth that makes it inefficient. In contrast to AlexNet, this VGGNet architecture contains numerous parameters. Moreover, VGGNet requires a lot of memory, which makes it more expensive computationally. In spite of being a deep network, and having huge complexity in computing, the model outperforms AlexNet and GoogLeNet in terms of productivity. Moreover, it is very simple to put into action. The ILSVRC-2014 challenge on ImageNet, for 1000 classes, scored a 92.70% precision rate.

### 3.6. ResNet

**Figure 5:**
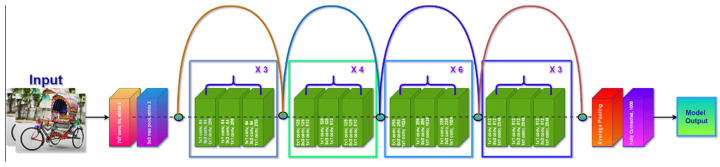
A pre-trained ResNet-50 network with residual connections.

The residual network (ResNet) is one of the most widely utilized CV architectures [20]. There are many benefits of using ResNet; the prominent benefit is that it can resolve the difficulty of degrading accuracy and the vanishing gradient by familiarizing the idea of shorter links. As a result, it is adaptable, task-specific, and capable of preparing very deep learning algorithms. Residual nets on the ImageNet dataset [47] have a depth of about 152 layers; these are eight times the depth of VGG networks. These, however, have a decreased risk of complications. On the ImageNet test set, a collection of these residual nets achieves a 3.57% error rate. On the tasks of ImageNet detection, ImageNet localization, COCO detection, and COCO segmentation (“The COCO dataset is a large-scale object detection, segmentation, and labeling dataset published by Microsoft. COCO has several features. Object segmentation, recognition in context, superpixel stuff segmentation, 330 K images (>200 K annotated), 1.5 million object instances, 80 object categories, 91 stuff categories, 5 captions per image, 250,000 persons with coordinates. Machine Learning and computer vision developers frequently utilize the COCO dataset for various computer vision applications”), deep residual networks came in first. ResNet-50 features 48 convolutional layers, as well as one max pooling and one average-pooling layer. The overall number of weighted layers is 50, with a total of 25, 583, 592 trainable parameters. It can do 3.8 × 10^9^ floating-point computations. Figure 5 depicts the architecture of the original ResNet-50.

We utilized ResNet-50 as the basic methodology in our suggested model, which was pre-trained on the ImageNet dataset [45] for image classification. We moved the initial 49 layers of ResNet-50, which were kept frozen on the classification model, adopting transfer learning techniques [48]. All of the other layers may be categorized as learnt feature extraction layers, which produce bottleneck features as the activation maps. We train a 13-fully connected softmax using the bottleneck characteristics of our native vehicle images as inputs, since we have 13 classes, and then swap the 1000 fully connected softmax with our trained data, which can be seen in Figure 5.

### 3.7. Proposed Classification Model

**Figure 6:**
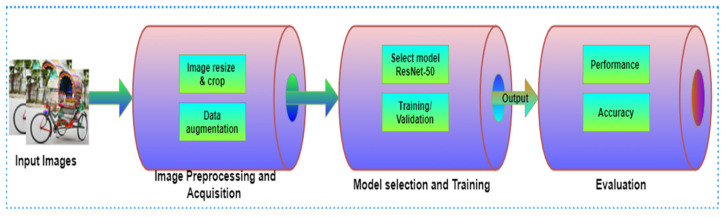
Proposed model approach pipeline.

We suggested a pipeline-based technique for our Bangladeshi native vehicle classification model for our DL model. The pipeline was divided into multiple phases, the first of which received images of Bangladeshi native vehicles from our Deshi-BD vehicle dataset, and the last of which classified the model. The output of each step was used as the input for the next stage. The suggested training method is divided into three stages: (i) image acquisition and processing; (ii) model selection and training; and (iii) evaluation. Figure 6 represents the suggested pipeline technique.

#### 3.7.1. Image Acquisition and Preprocessing

To create our Deshi-BD vehicle collection, we gathered images of Bangladeshi native vehicles. The dataset required preprocessing, image scaling, and appropriate conversions for the DL model. The amount of images in our sample were not the same size for all classes, which caused our dataset to be relatively unbalanced. To sidestep this problem, we used several augmentation approaches to increase the amount of images in our model, which allowed it to achieve greater generalization and recognition. After loading images into our chosen model from the Deshi-BD vehicle dataset, the images were first divided into training and validation data, according to the CNN architecture’s typical input size of normalized data to the size of 224 × 224 pixels.

#### 3.7.2. Model Selection and Training

A DL method takes, as input data, a map of characteristics to the target “X”, and predicts a model based on the output “Y”. ResNet-50 architecture was used for our model. During training, the algorithm optimized the parameters (update weights and biases) that were utilized for our model’s identification. In our experiment, we used 80% image data for our training model. Moreover, for validating the model, 20% image data were allocated to form a validation subset. On the test set, the performance of the suggested deep learning model was evaluated.

#### 3.7.3. Evaluation

Following the completion of our model’s training, the proposed DL model’s performance was assessed using a variety of assessment measures, including (i) accuracy; (ii) precision; (iii) recall; and (iv) $F_{1}-Score$.

### 3.8. Confusion Matrix as Evaluation Metrics

The confusion matrix is gathered in order to illustrate the predictions made by the developed framework on the testing data and to identify the bunch of frames incorrectly categorized. We investigated the correct execution of the native vehicle classification model in relation to model classifier indexes. The efficacy of our proposed native recognition and classification system is evaluated by generating evaluation metrics based on four major impacts used to test the classifier: true positives ($T_{p}$), true negatives ($T_{n}$), false positives ($F_{p}$), and false negatives ($F_{n}$). The overall acceptability of the fraction of the native vehicle classification model that is properly categorized is shown below:

Model accuracy ($A_{cc}$) improves the capacity to identify the Bangladeshi native vehicle categories properly. To assess the correctness of a testing dataset, we compute the percentage of true positive ($T_{p}$) and true negative ($T_{n}$) instances determined by given contacts:

(4) $$A_{cc}=\frac{T_{p}+T_{n}}{T_{p}+F_{p}+T_{n\;}+F_{n}}$$

Here, true positive $\left(T_{p}\right)$ shows the number of expected positive classes that are really positive classes, true negative $\left(T_{n}\right)$ counts the set of anticipated negative classes that are really negative classes, false positive $\left(F_{p}\right)$ displays the quantity of genuine negative classes anticipated as positive classes, false negative $\left(F_{n}\right)$ displays the quantity of real positive classes anticipated as negative classes. Additionally, model accuracy $A_{cc}$ is a ratio of appropriately predicted observations to all inspections. It is excellent when the datasets are symmetric.

Model precision $\left(P_{rc}\right)$ is the ratio of appropriately predicted observations of the total predicted positive assessment. Model precision $\left(P_{rc}\right)$ symbolizes the total amount of real positive class images among all classes images anticipated to be positive. It may be calculated as:

(5) $$P_{rc}=\frac{T_{p}}{T_{p}+F_{p}}$$

Model recall $\left(R_{ec}\right)$ is ratio of appropriately predicted clarifications to all assessments in the real class. The recall signifies all positive class image ratios, which are magnificently projected as positive. It may be calculated as:

(6) $$R_{ec}=\frac{T_{p}}{T_{p}+F_{n}}$$

$F_{1\;}-$*Score* is a harmonic mean of $P_{rc}\;\mathrm{and}\;R_{ec}$, and so it provides a combined knowledge about these two metrics. When the class distribution is imbalanced, $F_{1\;}-$ *Score* is more beneficial than accuracy. The $F_{1}\;-\;\mathrm{Score}$ is maximum when $\;P_{rc}$ and $R_{ec}$ are equal.

(7) $$F_{1}-Score=\frac{2\times P_{rc}\times R_{ec}}{P_{rc}+R_{ec}}\;$$

## 4. Result

### 4.1. Experiment Setup

The dataset-based platform configuration is used to test our proposed Bangladeshi native vehicle classification model. Our suggested tests were carried out on a heavy computing machine with an NVIDIA GeForce RTX2060 GPU, a DDR5 8 GB graphics processing unit (GPU), and a 64-bit Windows 10 operating system, with an Intel Core i7-8750H @ 2.2 GHz CPU and 16 GB RAM. We utilized Python 3.6 to develop our classification model, with the Keras Library being frontend and TensorFlow being backend for the framework.

### 4.2. Experimental Outline

**Figure 7:**
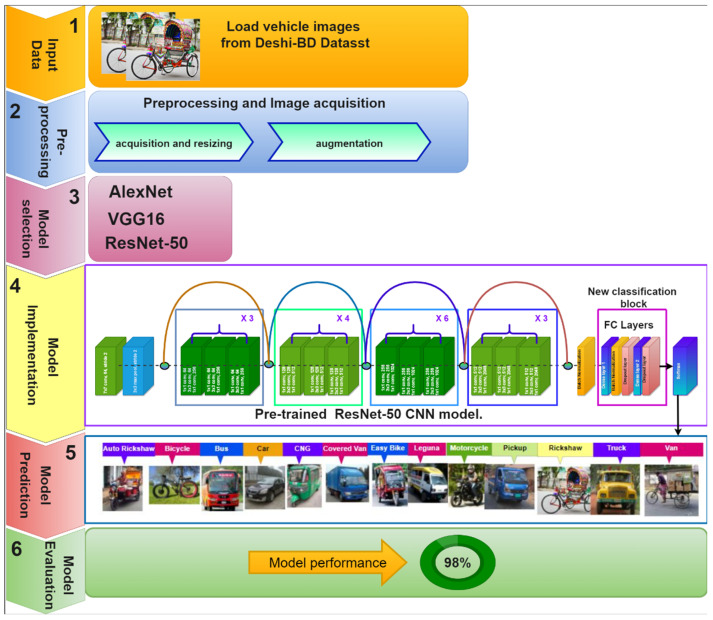
An overview of the proposed model outline.

We used the ResNet-50 model in the experimental framework to conduct Bangladeshi native vehicle classification. CNN architecture is one of the most innovative plans developed by He et al. [20], and it took top position in the ILSVRC-15 with a best-five inaccuracy ratio of 3.57% by demonstrating amazing results in object recognition and classification [34]. In early DL networks, growing network layers might cause a vanishing gradient issue, preventing the model from converging at its optimum. In the ResNet model, a unique skip connection-based method was presented, in which every input from the last layer was collected and delivered to the result of the following layer. Meanwhile, to reduce time complexity, a bottleneck suggestion was incorporated in the deeper network, driving this CNN model. We have been experimenting with a transfer learning approach in which a model trained for a fairly precise assignment may be modified to implement an alternative assignment by simply learning the new weights. In spite of having a smaller dataset, which is not sufficient for training from the beginning, this technique is highly effective. Figure 7 shows the overall framework of our customized DL model.

The Deshi-BD vehicle dataset was used to train ResNet-50 pre-trained architecture. A dropout layer is present at the bottom of the model to solve the vanishing gradient problem. A new, fully connected layer was added based on the classification block to conduct thirteen categories of native vehicle classifications, where each unit in the last layer was linked to the thirteen-class output probability using the softmax function. To confirm that these newly added layers learn greater-level feature characteristics from the dataset, we enlarged their learning rate in comparison to the prior layers’ learning rate. In addition, only the specific newly added layers included in the base model were trained, with the early convolutional layers frozen. The main concept behind freezing these layers was to accelerate convergence while avoiding the gradient outbreak throughout the training phase. After removing texture characteristics, classification was performed to compare the projected class to the real class. Over the training procedure, the network’s computation costs decreased, while the total trainable parameters of the modified CNN model also reduced. The architecture of our proposed ResNet-50 pre-trained CNN model is shown in Table 2.

In our experiment, we used a transfer learning approach on a pre-trained ResNet-50 architecture to recycle the weights of the network learnt from ImageNet, as shown in Figure 7. To avoid poor initialization, pre-trained weights were used in the model, as with its counterpart “random initialization of weights”. This network has 50 layers of depth, which was gained by substituting each 2-layer block in the innovative ResNet with the 3-layer bottleneck block [20]. This network’s input layer accepted red–green–blue (RGB) color images, reshaped to 224 × 224 pixels. To implement transfer learning, the network’s last fully connected layer, which performed ImageNet classification, was deleted. The pre-trained model’s early convolutional layers served as a foundation network for the newly adapted model. Following a universal mean pooling layer, two sets of batch normalization: (i) fully connected, and (ii) dropout layers, were added to the base network. The first fully connected layer has 512 neurons and the other has 256 neurons. Each fully connected layer was trailed by a ReLU activation layer. The training process of the pre-trained model is reduced by adding the batch normalization layers. The problem of overfitting was inherently reduced by the inclusion of global average pooling. The problem of overfitting in deep models typically fails to have a decent generalization on input that has not been seen earlier (test data). We employed numerous data augmentation techniques to prevent overfitting in our dataset. Finally, the final layer of our proposed model classified the Bangladeshi native vehicle images into thirteen classes using the softmax activation function.

**Figure 8:**
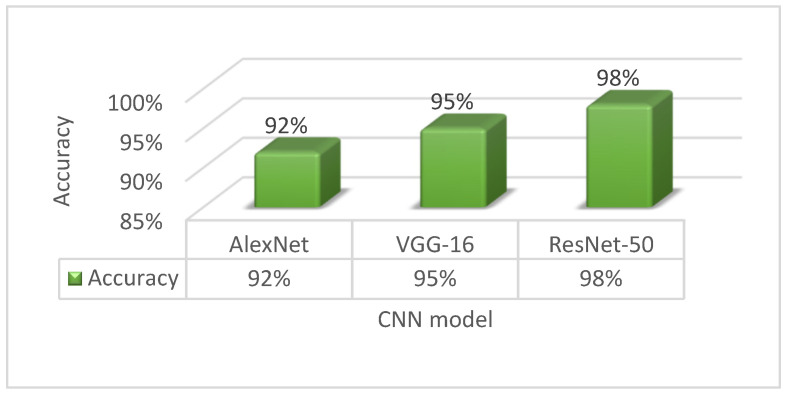
Validation accuracy of selected CNNs model.

TensorFlow resources were used to load the AlexNet, VGG-16, and ResNet-50 models for evaluation. TensorFlow was used to train all networks. In our proposed native vehicle classification model, the parameters are optimized using a stochastic optimization approach, called the Adam optimizer. We used dropout ratios of 0.50 for both dropout layers and set our learning rate at 0.0001. To train the model, we used a batch size of 32 and 50 epochs. Categorical cross-entropy, a commonly used loss function, was used to accumulate loss during the process, and validation of the network was performed after every epoch to evaluate the learning. Despite the fact that our proposed model was trained for 50 epochs, it obtained validation accuracy of 92% for AlexNet, 95% for the VGG-16 pre-trained model, and 98% for the ResNet-50 pre-trained model. Figure 8 displays the relative accuracy of various networks.

**Figure 9:**
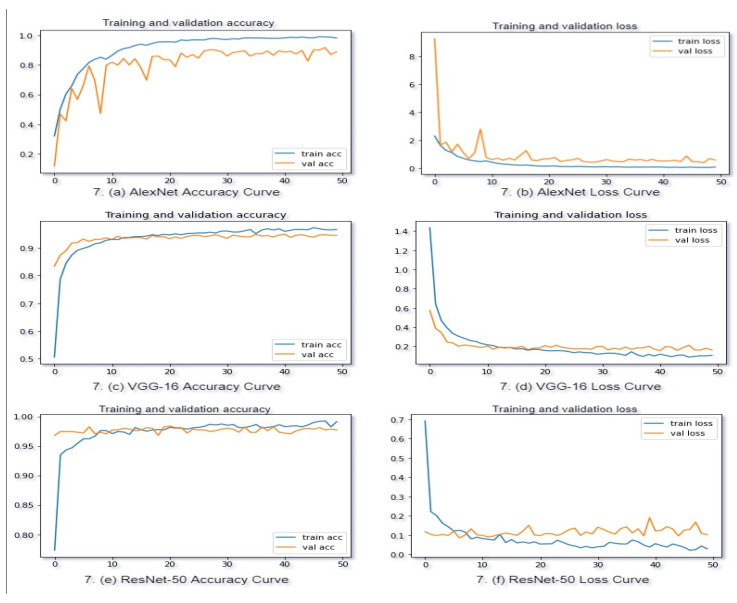
Accuracy and loss curve for training and validation.

In addition, for a better understanding of our approach, the learning process for both training and validation is illustrated using a loss and accuracy curve. We draw the loss and accuracy curves for each classification and recognition network model throughout the training phase. Figure 9 illustrates the model’s loss and accuracy values as a function of training epochs.

As seen above, the ResNet-50 model outperformed AlexNet and VGG-16 in terms of accuracy, with a 3% difference on average. As a result, after fine-tuning the design, ResNet-50 is expected to attain more accuracy.

### 4.3. Performance Analysis

**Figure 10:**
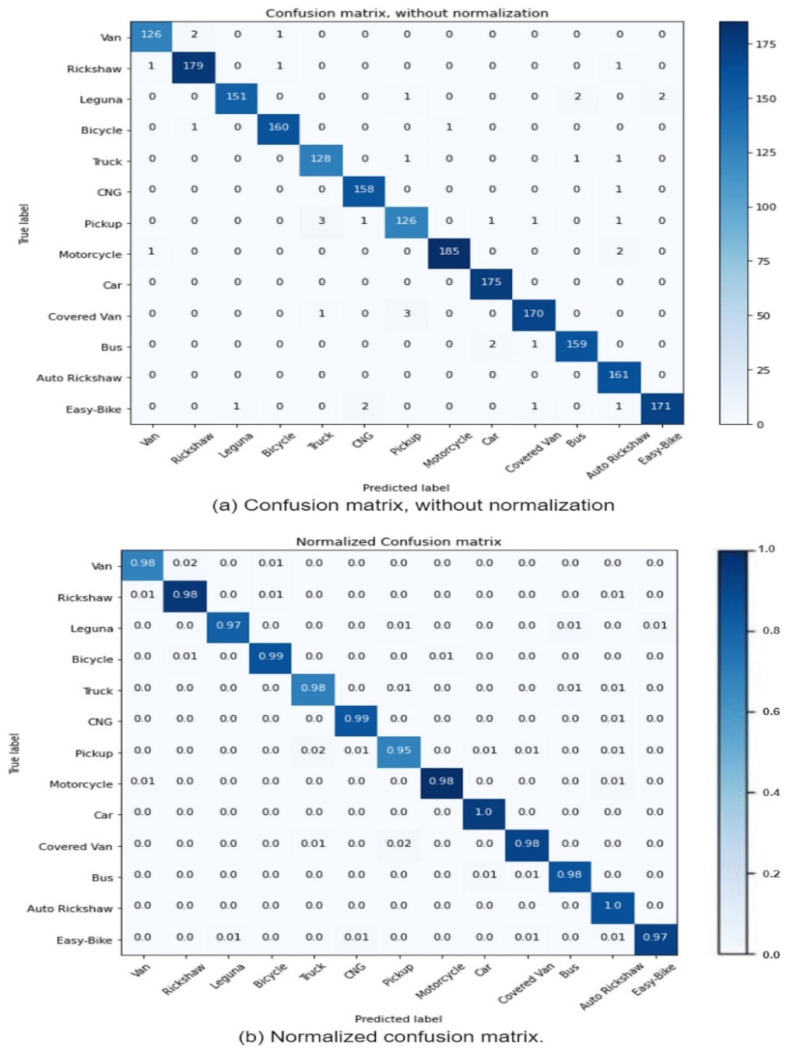
Confusion matrix for Deshi-BD native vehicle dataset; (**a**) confusion matrix, without normalization, and (**b**) normalized confusion matrix.

The confusion matrix is an excellent assessment directory for classification problems [49]. In a confusion matrix, rows and columns represent the real and projected classes. The recognition accuracy is represented in this matrix of prediction results by a blue color box, and the deeper the color, the more accurate the model recognition [50]. The horizontal axis represents the predicted values of the test set, such as auto rickshaw, bicycle, bus, car, CNG, cover van, easy bike, leguna, motorcycle, pickup, rickshaw, truck, and van. The vertical axis represents the real values of the test samples, such as auto rickshaw, bicycle, bus, car, CNG, cover van, easy bike, leguna, motorcycle, pickup, rickshaw truck, and van. The projected value of the model that is consistent with the true value of the test sample is located on the crosswise axis of the matrix. Figure 10 show the findings of our model’s confusion matrix.

In this part, we utilize a variety of evaluation parameters to assess the model classification outcomes, including precision, recall, and $F_{1}-Score$ results for our developed framework on the testing data. Equations (4)–(6) are used to calculate the outcome (Model Evaluation Metrics). Table 3 provides a thorough performance analysis of the classification impact based on each category. As it can be observed, the classification result of our chosen model had a 98% average precision, recall, and $\;F_{1}-Score.$

We saw that Figure 8 represents the comparative experiment findings in this study; moreover, we compare the findings with two other CNN models; AlexNet and VGG-16 pre-trained architecture. There has been a significant improvement in model performances in terms of accuracy and other assessment criteria. As shown in Table 4, our presented model outperformed the AlexNet and pre-trained VGG-16 models.

The AlexNet model only obtained a classification accuracy of almost 93%, while the VGG-16 model achieved a classification accuracy of nearly 95%, which was around 6% and 3% lower than the accuracy attained by our suggested ResNet-50 pre-trained model, respectively. Aside from that, the most probable reason seemed to be that our suggested ResNet-50 pre-trained model had less trainable parameters than VGGNet (134 M), and VGGNet did not include skip connections to make calculations easier. Built on the findings, we can conclude that our suggested model, which is based on the residual network ResNet-50, is more robust for the Bangladeshi native vehicle classification-based CNN model that can extract robustness for recognition and classification. As can be observed, our proposed ResNet-50 pre-trained DL model, based on transfer learning, outperforms the other three classification methods, in terms of classification accuracy.

## 5. Discussion

The proposed Bangladeshi native vehicle classifier was related to the existing vehicle classifiers [16,51,52,53] to verify the selected network’s efficacy. The existing networks have been reproduced on the suggested database. The GoogLeNet architecture-based vehicle classification method and the 22-layer depth network, was presented by Zhuo et al. [51]. Gao et al. [52] developed an AlexNet that focuses on vehicle classifier using five convolutional layers as well as three fully connected layers. Additionally, an inception architecture-based classifier was suggested by Zakria et al. [16]. A self-proposed CNN-based vehicle classifier with 13 convolutional layers, as well as one fully connected layer, max-pooling, as well as dropout layers, is followed in the model, which was presented by Shivai et al. [53]. All of these models established good performances on their datasets. However, these existing systems [16,51,52,53] did not focus on massive data because they are constructed of many smaller depth networks and are developed on narrow categories that do not include road transport mobility. Table 5 compares this study to other baseline techniques.

As a result, for the native vehicle classification problem, the above techniques perform poorly in real-time classification purposes. Furthermore, it is crucial to note that these methods are trained on imbalanced data, which is a key element in the true presentation of local vehicle classification architecture. As a result, the achievement of the current models is discriminating when assessing the presented Deshi-BD stable database. However, our proposed native vehicle classification network was trained on the own-built Deshi-BD native vehicle database, which covers thirteen common road traffic classes in Bangladesh, with 10,440 images. As a result of evaluating all of the aforementioned systems, we conclude that our suggested native vehicle classifier outperformed the other existing vehicle classifiers, in terms of accuracy. Additionally it has greater generalization capabilities for taking a broader variety of information, as well as adaptability to South Asian traffic surveillance applications. In the future, we hope to expand our research and develop fine-grained classification systems that will increase the usefulness of the proposed approach in ITS.

## 6. Conclusions

Road traffic accidents are major causes of death and injury across the world, which is a concerning problem. Thousands of individuals are killed or seriously disabled in traffic crashes every year. When compared to other developing countries, Bangladesh has a much higher percentage of death and injury from traffic accidents. To avoid this serious problem, in this paper, we presented a customized DL model-based method to detect and classify Bangladeshi native vehicles, to develop the effectiveness of ITS. In our work, for the training classification algorithm, a new dataset was introduced for Bangladeshi native vehicles, namely the Deshi-BD dataset, having 10,440 images with thirteen categories to train the classification system. At first, to validate the performance of our Deshi-BD vehicle dataset, three advanced CNN architectures, AlexNet, VGG, and ResNet, were trained. The ResNet-50 model was constructed based on transfer learning for the Bangladeshi native vehicle classification. Transfer learning was used to improve the ResNet-50 architecture by adding a new classification layer to the original network. In our model, we also evaluated the performance by using various attributes, such as (a) accuracy, (b) precision, (c) recall, and (d) $F_{1}-Score$. We also compared our model with VGGNet and AlexNet. Results from our study exposed that our recommended native vehicle classification system achieved accuracy of 98% on the Deshi-BD dataset, which is significantly greater than other existing advanced classification systems. This methodology proved to be “vigorous” while deviations, such as vehicle size, vehicle shade, position, and weather, were considered. Moreover, we proposed native vehicle classification methods that utilize fewer parameters, so that training costs can be lower and, thus, have the prospective to support intelligent traffic management technologies. Finally, we conclude that our approach outperforms other current methods in vehicle type classification under all conditions.

However, in order to improve our research, we need to gather relevant data and evaluate other vehicles that are also visible on the road. Our future work will involve adding more types of native vehicle images to the dataset, as well as further adjusting the network topology and parameters for vehicle classification. We plan to expand our model in real-time detection and tracking for smart traffic monitoring system. We also plan to grow our system to include vehicle counting, automatic license plate recognition, and traffic congestion detection modules before combining them into a full autonomous traffic monitoring system.

## Figures and Tables

**Table 1:** Data description of Deshi-BD vehicle dataset.

No.	Vehicle Classes	Data Augmentation	Total Image
1	Auto Rickshaw	40	800
2	Bicycle	15	805
3	Bus	0	800
4	Car	20	865
5	CNG	15	830
6	Covered Van	30	810
7	Easy Bike	50	810
8	Leguna	0	760
9	Motorcycle	80	870
10	Pickup	20	740
11	Rickshaw	90	890
12	Truck	20	720
13	Van	0	740
	Total	380	10,440

**Table 2:** Architecture of our proposed ResNet-50 pre-trained CNN model.

Layer Name	Output Size	Layers
Conv1 Pooling	112×112	7×7,64, stride 2 3×3maxpool, stride 2
Conv2	56×56	$\left[\begin{array}{c} 1\times 1,\;64\\ 3\times 3,\;64\\ 1\times 1,\;256 \end{array}\right]\times 3$
Conv3	28×28	$\left[\begin{array}{c} 1\times 1,\;128\\ 3\times 3,\;128\\ 1\times 1,\;512\; \end{array}\right]\times 4$
Conv4	14×14	$\left[\begin{array}{c} 1\times 1,\;128\\ 3\times 3,\;128\\ 1\times 1,\;512\; \end{array}\right]\times 6$
Conv5	7×7	$\left[\begin{array}{c} 1\times 1,\;128\\ 3\times 3,\;128\\ 1\times 1,\;512\; \end{array}\right]\times$3
Pooling Our implemented classification block	1×1	Adaptive average pooling 2D fc1:Input feature=1024, Output feature=512 ReLU (in place), dropout=0.5 fc2:Input feature=512, output feature=13 Softmax( ) Classification output = (loss =‘categorical_crossentropy’, optimizer = ‘adam’, metrics = (‘accuracy’))

**Table 3:** The classification report for our proposed ResNet-50 pre-trained model.

Vehicle Type	Precision	Recall	$F_{1}-Score$	Number of Test Images
Van	0.98	0.98	0.98	129
Rickshaw	0.98	0.98	0.98	182
Leguna	0.99	0.97	0.98	156
Bicycle	0.99	0.99	0.99	162
Truck	0.97	0.98	0.97	131
CNG	0.98	0.99	0.99	159
Pickup	0.96	0.95	0.95	133
Motorcycle	0.99	0.98	0.99	188
Car	0.98	1	0.99	175
Covered Van	0.98	0.98	0.98	174
Bus	0.98	0.98	0.98	162
Auto Rickshaw	0.96	1	0.98	161
Easy Bike	0.99	0.97	0.98	176

**Table 4:** Report on comparative performance analysis.

Model Type	Precision	Recall	$F_{1}-Score$	Accuracy
AlexNet	0.915	0.935	0.935	0.928
VGG-18	0.942	0.945	0.951	0.946
ResNet-50	0.979	0.981	0.981	0.98

**Table 5:** A comparison of the proposed approach to existing vehicle classifiers.

Authors	Features	Model	Accuracy (%)
Zhuo et al. [51]	GoogLeNet based	GoogLeNet	95.49
Gao et al. [52]	AlexNet based	AlexNet	92.61
Shivai et al. [53]	CNN based	CNN	88.96
Zakria et al. [16]	Inception based	Inception	92.77
Our proposed model	ResNet-50 based	Pre-trained model	98

## Data Availability

Not applicable.
